# Delayed Reduction in the Left Ventricular Outflow Tract Gradient After Endocardial Radiofrequency Ablation for Septal Hypertrophy in a Patient With Hypertrophic Obstructive Cardiomyopathy: A Case Report

**DOI:** 10.7759/cureus.87011

**Published:** 2025-06-30

**Authors:** Hongshuo Chu, Ting Gao, Ye Zhu, Huayun Wang, Zerui Hu, Jingyuan Ni, Zixiu Wei

**Affiliations:** 1 Department of Cardiology, Institute of Cardiovascular Diseases of Jining Medical Research Academy, Jining First People's Hospital, Jining, CHN; 2 Department of Rehabilitation Medicine, Jining First People's Hospital, Jining, CHN; 3 Department of Psychiatry, Shandong Daizhuang Hospital, Jining, CHN; 4 Department of Ultrasonic Medicine, Jining First People's Hospital, Jining, CHN

**Keywords:** case report, hypertrophic obstructive cardiomyopathy, intracardiac echocardiography, myocardial edema, radiofrequency ablation, transthoracic echocardiography, ventricular outflow tract gradient

## Abstract

We report a case of a 79-year-old female with hypertrophic obstructive cardiomyopathy (HOCM) who demonstrated a delayed reduction in the left ventricular outflow tract (LVOT) gradient following endocardial radiofrequency ablation of septal hypertrophy (ERASH). The patient presented with diffuse interventricular septal thickening and systolic anterior motion (SAM) of the anterior mitral valve leaflet, with a markedly reduced six-minute walking distance of less than 50 meters. To minimize procedural risks, ERASH was performed using the CARTO3 (Biosense Webster, Inc., Irvine, California, USA) and CARTOSOUND systems (Biosense Webster, Inc., Irvine, California, USA). Intraoperatively, SAM resolved and the LVOT gradient showed an immediate decline (93 mmHg to 43 mmHg). The patient experienced gradual improvement in functional status and remained asymptomatic post-procedure. Interestingly, four days post-procedure, follow-up transthoracic echocardiography (TTE) demonstrated a delayed, further reduction in the LVOT gradient.

This case highlights the potential for delayed hemodynamic improvement following ERASH, potentially attributable to transient post-ablation myocardial edema, as evidenced by the increase in end-diastolic interventricular septum thickness detected by TTE. Further studies are needed to assess procedural factors such as ablation power settings, ablation lesion size, and age-related myocardial degeneration.

## Introduction

Endocardial radiofrequency ablation of septal hypertrophy (ERASH) is a safe, minimally invasive percutaneous procedure for the treatment of hypertrophic obstructive cardiomyopathy (HOCM). Compared to surgical myectomy and alcohol septal ablation, ERASH offers distinct advantages, including reduced procedural trauma and more flexible technical requirements. These characteristics make it particularly suitable for elderly HOCM patients, who may be at a higher surgical risk [1]. The left ventricular outflow tract (LVOT) gradient - representing the systolic pressure difference between the left ventricle and aorta - and systolic anterior motion (SAM) of the mitral valve - a hallmark echocardiographic feature of dynamic LVOT obstruction - serve as critical diagnostic markers in HOCM. However, to our knowledge, a delayed reduction in LVOT gradient following ERASH has not been previously reported.

In 2021, Lawrenz et al. conducted a study involving 41 patients who underwent ERASH, highlighting its suitability for elderly HOCM patients due to minimal procedural trauma [1]. This builds upon their earlier work: in 2004, they first reported the efficacy of ERASH in a case study, documenting a dramatic reduction in LVOT gradient post-procedure [2]. Later, in 2011, their retrospective analysis of 19 HOCM patients further reinforced these findings, showing consistent and rapid LVOT gradient improvement by echocardiography in all cases [3]. In the same year, to our knowledge, Sreeram et al. published the first documented case linking myocardial edema to ERASH, reporting a fatal outcome in a 4.6-year-old child with HOCM. The patient developed acute left ventricular dysfunction secondary to paradoxical LVOT obstruction exacerbation, with intracardiac echocardiography (ICE) confirming tissue edema at ablation sites [4].

Since then, many ERASH procedures have been guided by ICE. In 2016, Cooper et al. described a similar phenomenon of LVOT gradient elevation leading to pulmonary edema, though imaging confirmation was unavailable in this case [5]. A report highlighted the use of advanced measurement methods, including invasive hemodynamics with CARTO3 mapping (for real-time 3D electroanatomic reconstruction; Biosense Webster, Inc., Irvine, California, USA) and CARTOSOUND systems (for ultrasound-based anatomical visualization; Biosense Webster, Inc., Irvine, California, USA), which facilitated more precise pressure measurement and ablation [6]. According to a recent study on ERASH, paradoxical LVOT gradient increases were observed in 9% of HOCM patients undergoing ERASH [1].

Herein, we present a rare case demonstrating a delayed reduction in the LVOT gradient four days after an initial decrease following ERASH, guided by electroanatomic mapping and ICE.

A previous version of this article was posted on ResearchSquare on August 25, 2023.

## Case presentation

**Figure 1:**
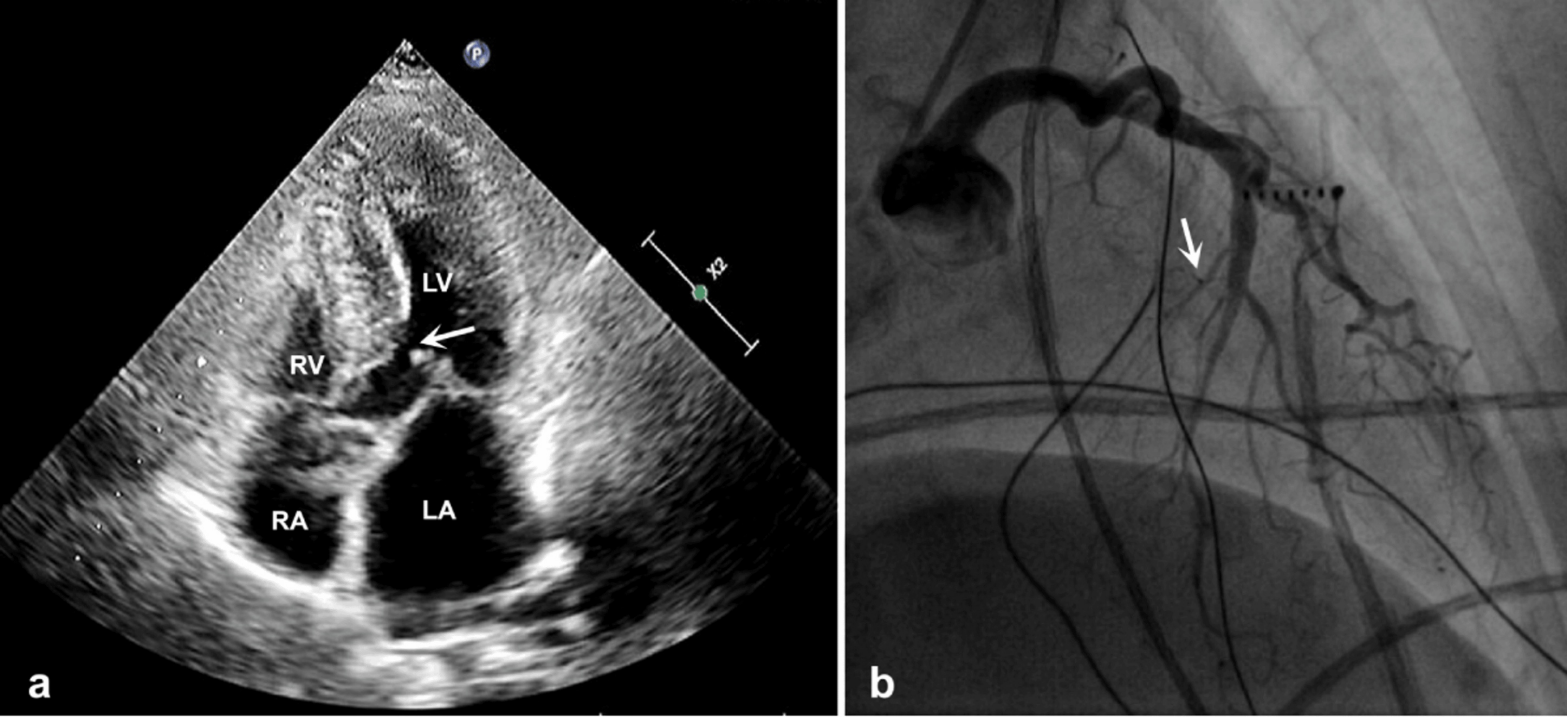
TTE and left coronary angiography before ablation a) TTE shows septal thickening and SAM (white arrow) of the anterior mitral valve leaflet in the apical four-chamber view, in systole. b) Moderate stenosis and small septal branches (white arrow) are seen in the mid-portion of the anterior descending branch. TTE, transthoracic echocardiography; SAM, systolic anterior motion; LV, left ventricle; RV, right ventricle; LA, left atrium; RA, right atrium

A 79-year-old woman with a history of hypertrophic cardiomyopathy presented with exacerbated stuffiness and wheezing. After being diagnosed with moderate-to-severe stenosis and obstruction in the LVOT two years ago, the patient took oral metoprolol and diltiazem to relieve her symptoms. She had a history of bronchial asthma and lumbar spine disease. Smoking and drug abuse were denied. Physical examination revealed a blood pressure of 135/85 mmHg, a heart rate of 94 bpm with a sinus rhythm, and a systolic murmur along the left sternal border. A six-minute walk test revealed a walking distance of less than 50 meters. Laboratory evaluation on admission showed a normal troponin I concentration, an elevated N-terminal pro-brain natriuretic peptide at 5093 pg/mL (normal range: 12-125 pg/mL), and an elevated low-density lipoprotein cholesterol at 5.41 mmol/L (normal range: 0-3.88 mmol/L). Chest radiography and electrocardiography were unremarkable. Transthoracic echocardiography (TTE) demonstrated diffuse thickening of the interventricular septum and SAM of the anterior mitral valve leaflet (Figure 1a). Despite up-titration of medical therapy, the symptoms of left-sided heart failure persisted.

**Figure 2:**
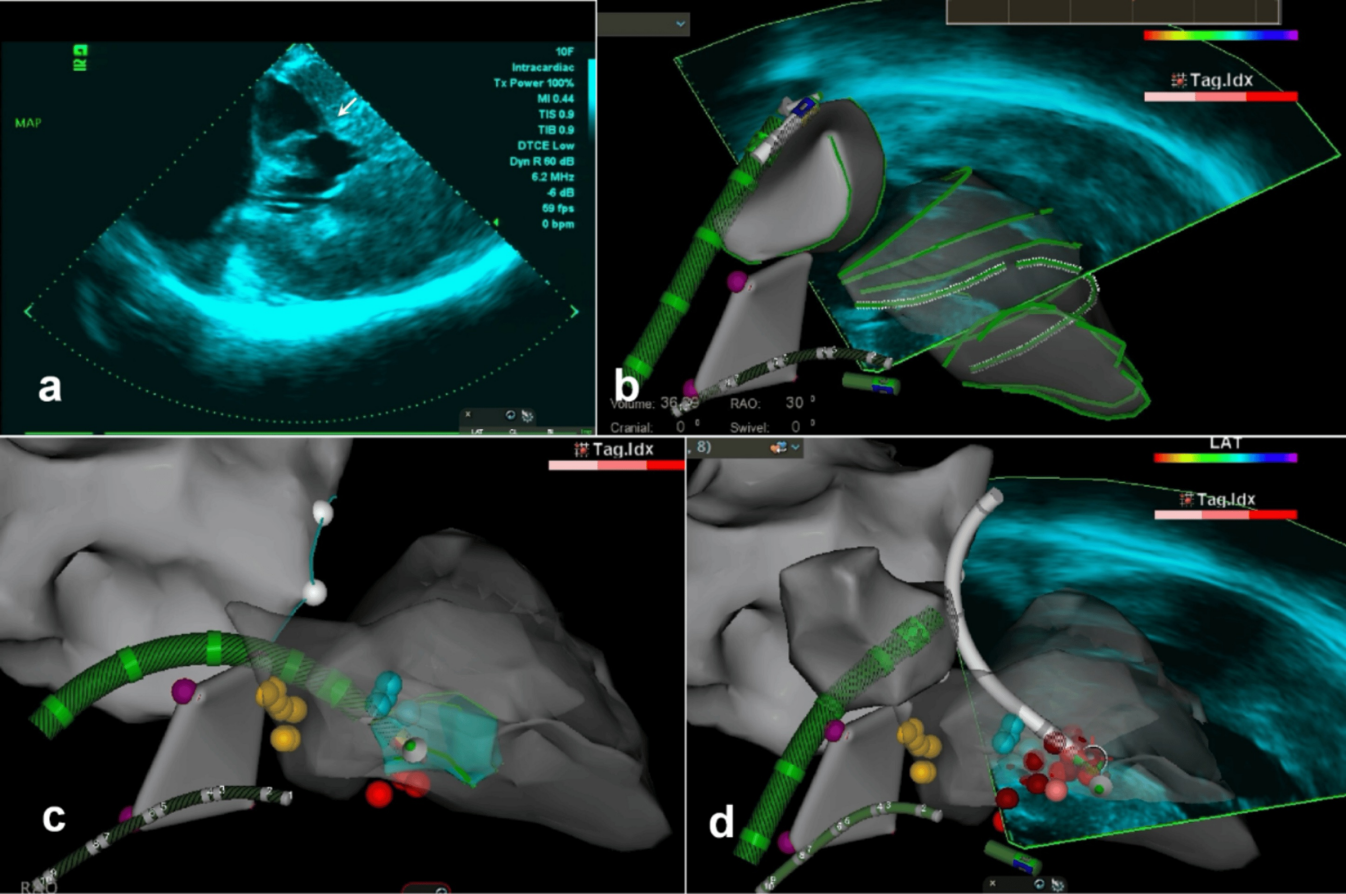
ICE-guided ERASH with the CARTO3 and CARTOSOUND system a) SAM (white arrow) on ICE; b) left ventricular mapping procedure; c-d) the ablation procedure was performed using both retrograde transaortic and transseptal approaches. The red dots show the ablation position; the yellow dots show the bundle of His; the purple dots show the tricuspid annulus; and the white dots show the mitral annulus. ICE, intracardiac echocardiography; ERASH, endocardial radiofrequency ablation for septal hypertrophy

Considering the extensive trauma and prolonged recovery, the patient declined surgical myectomy. Coronary angiography revealed both the small size and absence of an ideal distribution of the septal branch within the hypertrophic septum, which rendered alcohol delivery ineffective (Figure 1b). Following team discussion, we adopted an optimized approach utilizing ERASH with ICE guidance to achieve targeted septal thinning. This strategy was chosen to minimize procedural invasiveness and enhance time efficiency compared to conventional septal reduction options, such as surgical myectomy or alcohol septal ablation. The procedure was performed via the right femoral artery and the bilateral femoral veins. ICE was introduced using a 10-pole coronary sinus catheter, while an angled pigtail catheter invasively measured the peak resting LVOT gradient (93 mmHg; pre-procedural TTE: 62 mmHg). Using the CARTO3 mapping and navigation system, the ICE catheter probe was positioned in the right atrium. Left-sided visualization was achieved using rotational and transseptal ICE imaging. A three-dimensional reconstruction of the ventricular septum was created by integrating ICE images with the CARTOSOUND system (Figure 2a). Additionally, SAM was detected by ICE, and the septal ablation target area was identified (Figure 2b). The bundle of His and bundle branch potentials were identified on the left side of the septum using a radiofrequency ablation catheter (Thermocool SF; Biosense Webster, Inc., Irvine, California, USA) to prevent conduction block during ablation. To ensure stable catheter contact during ablation of this extensive target area, we employed both retrograde and transseptal access routes. The ablation catheter was advanced to the septum via the transfemoral arteries and atrial septum (Figures 2c-2d). We performed ablation at the maximal contact area between the basal septum and the mitral valve, as well as at the thickest region of the septum, at a power of 50 W and a temperature of 30℃. After 39 minutes of ablation, SAM disappeared, and ICE revealed a hyperechoic region corresponding to the ablated, thinner septum. The resting LVOT gradient decreased to 43 mmHg, indicating a successful procedure. Despite the patient’s initial anxiety, the intervention was well tolerated and completed without complications. Improvements in cardiac functional status were primarily assessed using the six-minute walk test; however, detailed data regarding symptom scoring or additional objective tests (e.g., exercise echo) were not available.

The patient demonstrated rapid and sustained symptomatic improvement, with complete resolution of chest tightness and wheezing. Twenty-four hours post-procedure, the patient was able to walk 100 meters in the six-minute walk test. However, TTE performed on postoperative day 4 revealed an elevation in the resting LVOT gradient and a thicker end-diastolic interventricular septum (Table 1), likely due to post-ablation edema or procedural inflammation. Despite this, her exercise capacity continued to improve without recurrence of symptoms. By the two-month follow-up, most parameters had significantly improved compared to day 4, including NYHA (New York Heart Association) class, septal thickness, resting LVOT velocity, and gradient. Concurrently, the patient walked over 300 meters in the six-minute walk test without discomfort. The patient declined cardiac magnetic resonance imaging (MRI) and cardiopulmonary exercise testing due to advanced age and the complexity of the examinations. The timeline of historical and current information from this episode of care is presented in Table 2 (see Appendix).

**Table 1:** Cardiac function and transthoracic echocardiography before and after ERASH Note: Pre-procedure and follow-up LVOT gradients were assessed by TTE; intraprocedural gradients were measured invasively during ERASH. The patient declined NT-proBNP testing at postoperative day 4 and the 18-month follow-up. LVOT, left ventricular outflow tract; NT-proBNP, N-terminal pro-brain natriuretic peptide (NT-proBNP levels were not measured at post-operative day 4 or 18 months, as blood tests were declined by the patient during those times); NYHA, New York Heart Association; SAM, systolic anterior motion; ERASH, endocardial radiofrequency ablation for septal hypertrophy

Clinical Investigations	Before ERASH	4 days after ERASH	2 months after ERASH	18 months after ERASH
Walking distance, six-minute walk test (m)	<50	100	>300	>300
NT-proBNP (pg/mL)	5093	-	2576	-
NYHA functional class	IV	III	II	I
End-diastolic interventricular septum thickness (mm)	20	22	15	14
SAM	Yes	No	No	No
Peak LVOT flow velocity at rest (m/s)	3.9	3.8	2.3	2.3
Peak LVOT gradient at rest (mmHg)	62	58	22	21

## Discussion

HOCM typically presents with more severe symptoms than the non-obstructive form, including exertional dyspnea, chest pain, palpitations, and syncope. TTE is the primary imaging modality for identifying interventricular septal hypertrophy and SAM. The reduction in cardiac output caused by LVOT obstruction increases the risk of heart failure, atrial fibrillation, and sudden death [7].

Although septal myectomy is effective in alleviating symptoms, its invasive nature and prolonged convalescence often make it less favorable for older patients, who tend to have lower expectations and tolerance for surgical intervention [8,9]. Therefore, interventional therapy is more suitable for elderly patients. Alcohol septal ablation was considered in the present study based on the presence of ideal coronary septal branches, which met the criteria, including typical anatomical distribution, adequate perfusion territory, and favorable angulation for guidewire access. However, previous studies have reported that 5%-7% of patients with HOCM have atypical vascular anatomy or perfusion patterns that preclude effective alcohol delivery [5,10,11]. ERASH has progressively emerged as a promising alternative for patients with HOCM who are not suitable candidates for surgical myectomy or alcohol septal ablation [12]. ERASH has been shown to consistently reduce the LVOT gradient, ameliorate symptoms, and enhance cardiac function [3,5,13]. In most patients, the gradient reduction occurs shortly after the ERASH procedure. Nevertheless, a paradoxical increase in the LVOT gradient has been observed in certain age groups [3,6,14].

From 2004 to 2021, Lawrenz et al. conducted three pivotal studies on ERASH. The earliest case documented a significant and sustained reduction of LVOT gradient during ablation [2]. In a 2011 study involving 19 patients with HOCM, various access approaches were utilized, including the inferior vena cava route for right ventricular ablation, the retrograde transaortic approach, and transseptal puncture for left ventricular ablation. Notably, eight patients had previously undergone alcohol septal ablation prior to their inclusion in the study [3]. In 2021, Lawrenz et al. published a comprehensive summary of their 17-year institutional experience with ERASH in 41 HOCM patients. They emphasized a significant observation: a paradoxical increase in obstruction, likely induced by notable myocardial edema associated with high-power (>60 W) ablation, was identified as a critical complication [1]. Additionally, a retrospective study involving 32 children with HOCM reported one death due to paradoxical worsening of LVOT obstruction [4]. Two patients in separate studies developed pulmonary edema immediately following ablation. In one case, the condition resolved with ventilatory support and intravenous diuretics, while the other case was managed with reintubation, intravenous dexamethasone, and right ventricular apical pacing [5,15].

In recent studies, the use of electroanatomic mapping systems and an irrigated-tip ablation catheter has become increasingly common in ERASH procedures. When combined with ICE, these technologies allow for precise navigation and accurate placement of the radiofrequency ablation catheter at the targeted myocardial site [5]. In our case, the use of ICE and the CARTOSOUND mapping system facilitated precise visualization of the septal anatomy and ablation target, allowing for efficient ablation and potentially reducing the risk of complications, which may have contributed to the observed safety and immediate symptom improvement. However, the observation of symptom improvement despite an initially unchanged LVOT gradient is unexpected and, to our knowledge, has not been previously reported following ERASH.

Interestingly, TTE on postoperative day 4 showed that the left ventricular septal thickness, LVOT gradient, and flow velocity remained virtually unchanged compared to pre-procedural measurements (Table 1). We propose that the resolution of systolic SAM contributed to decreased ventricular stiffness and improved cardiac function. A distinct echogenic strip was observed on the surface of the thinned septum, confirming the efficacy of ablation. However, the unchanged LVOT gradient was likely attributable to post-ablation myocardial edema, as previously described. In comparison to cases with paradoxical increase in LVOT obstruction or pulmonary edema, our patient experienced a prompt alleviation of symptoms with an immediate reduction of LVOT gradient at rest, measured by invasive catheter. We hypothesize that the use of ICE and limiting the ablation power to <60 W may have resulted in only mild myocardial edema, contributing to a gradual rather than immediate reduction in the LVOT gradient. This contrasts with previous reports of paradoxical increases in gradient associated with higher power settings (>75 W) [1]. However, this remains a hypothesis requiring further validation. Although direct evidence is lacking, we postulate that myocardial aging and fibrosis may alter the tissue response to thermal injury, potentially affecting lesion formation, edema resolution, and remodeling after ablation. Badger et al. investigated acute and chronic myocardial changes following ablation and found that enhancement patterns observed on cardiac MRI at 24 hours reflected a transient inflammatory response, which resolved before scar formation at 30 days [16]. These findings provide a potential mechanism for delayed reduction.

This case report is subject to several limitations. Cardiac MRI could have provided definitive evidence of post-ablation myocardial edema, but the patient declined this examination. Invasive hemodynamic assessment during follow-up was avoided to minimize procedural trauma. Additionally, exercise testing and provocation assessments were not performed, as the procedure duration was intentionally shortened to reduce risk. These limitations hinder the full evaluation of the delayed gradient response. Serial imaging and functional testing in future studies may provide further insights. The potential impact of the patient's anxiety on the measurement of the gradient was not considered. To minimize trauma, invasive hemodynamic assessments were not performed during follow-up. Furthermore, data on LVOT gradient following provocation or exercise were not collected, as the procedure time was intentionally reduced to mitigate risk.

## Conclusions

To our knowledge, a delayed reduction in LVOT gradient following ERASH has not been previously reported in HOCM patients. This case suggests that mild myocardial edema - potentially influenced by lower ablation power (<60 W) or patient-specific myocardial characteristics - may contribute to this delayed improvement. Despite this, the patient showed symptomatic relief, no complications, and sustained gradient reduction during follow-up. Our findings raise questions about relying solely on immediate post-procedure gradient reduction as an endpoint. Further studies are needed to clarify the relationships among ablation power, gradient dynamics, and myocardial edema in optimizing ERASH outcomes.

## Appendices

**Table 2:** Historical and current information from this episode of care organized as a timeline TTE, transthoracic echocardiography; LVOT, left ventricular outflow tract; ERASH, endocardial radiofrequency ablation for septal hypertrophy; SAM, systolic anterior motion

Time point		Clinical findings/observations
2 years before hospitalization		TTE revealed moderate-to-severe stenosis and obstruction in LVOT.
Before ERASH		The patient presented in our hospital with exacerbated stuffiness and wheezing. TTE demonstrated diffuse thickening of the interventricular septum and SAM of the anterior mitral valve leaflet (Figure 1a).
During ERASH		Ablation at the maximal contact area between the basal septum and the mitral valve, and at the thickest part of the septum, was performed at a power of 50 W and a temperature of 30℃. SAM disappeared, and a hyperechoic strip on the surface of the thinner septum was revealed. Reduction of the LVOT gradient following the procedure indicated a successful outcome.
After ERASH	24 hours	We observed improvement in the 6-minute walk test and relief of symptoms.
	4 days	The patient demonstrated a continuous increase in exercise capacity without any symptoms. The LVOT gradient and flow velocity at rest on TTE demonstrated a delayed reduction (Table 1).
	2 months	We observed further improvement in the 6-minute walk test and a notable reduction in LVOT gradient.
	18 months	The ablation efficacy achieved long-term stability.
